# Supplementary material for: Intermittent dynamics in complex systems driven to depletion
Source: Sci Rep. 2018 Mar 19;8:4825. doi: 10.1038/s41598-018-23033-x (PMC5859173; doi:10.1038/s41598-018-23033-x)
Supplement: Supplementary file 1 — supplementary information [file 41598_2018_23033_MOESM1_ESM.pdf]

Intermittent dynamics in complex systems driven to depletion

Juan V Escobar[1,*] & Isaac Pérez Castillo[2,3]

[1] *Instituto de Física, Universidad Nacional Autónoma de México. Apdo. Postal 20-364, Cd. Mx., Mexico, C.P. 04510*

[2] *Departamento de Cuántica y Fotónica, Instituto de Física, Universidad Nacional Autónoma de México. Apdo. Postal 20-364, Cd. Mx., Mexico, C.P. 04510*

[3] *London Mathematical Laboratory, 14 Buckingham Street, London WC2N 6DF, United Kingdom*

1 Analytical treatment of the model

In this section we show the derivation of a naïve mean field solution for the dynamics. Based on this result we perform a bifurcation type analysis to obtain the line separating the synchronous regime to the asynchronous one,

A naïve dynamical mean-field equation. As explained in the main text the microscopic dynamics is given by

$$\sigma_i(t+1) = \sigma_i(t)\Theta\left(T_i(t) - x_i\right) \,, \tag{1}$$

with $N(t) = \sum_{i=1}^{N} \sigma_i(t)$ and $\sigma_i(t) \in \{0,1\}$ and $q(t) = \sum_{i=1}^{N(t)} T_i(t)$. The probability of finding the local attendances at a given configuration $\{T_i(t)\}$ is a multinomial distribution with uniform

distirbution $1/N(t)$, that is:

$$\mathrm{Prob}(\{T_i(t)\}) = \frac{q(t)!}{\prod_{i=1}^{N(t)} q_i(t)!}\left(\frac{1}{N(t)}\right)^{q(t)} . \quad (2)$$

Thus, the first two cumulants of $T_i(t)$ are $\mathrm{E}[T_i(t)] = q(t)/N(t)$ and $\mathrm{Var}[T_i(t)] = q(t)[N(t) - 1]/N^2(t) \simeq q(t)/N(t)$, respectively. Moreover the covariance is $\mathrm{Cov}[T_i(t), T_j(t)] = -q(t)/N^2(t)$, which implies that for large $N(t)$ the variables are weakly correlated. This suggests to reasonably assume the occupancies $\{T_i(t)\}$ to be i.i.d random Gaussian variables as follows :

$$T_i(t) = \frac{q(t)}{N(t)} + \sqrt{\frac{q(t)}{N(t)}}\zeta_i(t) , \quad (3)$$

with $\zeta_i(t) \sim \mathcal{N}(0,1)$. Since the occupancies are definite positive, one can introduce a cut-off in the Gaussian distribution to ensure that $T_i(t) \geq 0$, However this only affects the dynamics at the late stages, so one can simply ignore it for the sake of simplicity. Similarly, we assume that the distribution of thresholds follows a Gaussian distribution (same argument for a possible cut-off) with mean value μ and variance σ^2, or in other words: $x_i = \mu + \sigma\xi_i(t)$ with $\xi_i(t) \sim \mathcal{N}(0,1)$. Then, starting from the microscopic dynamical equation (1), we try to write a corresponding closed evolution equation for the variable $N(t) = \sum_{i=1}^{N} \sigma_i(t)$ neglecting time and space fluctuations (a naïve mean field approximation), *viz.*

$$\begin{aligned} N(t+1) &= \sum_{i=1}^{N} \sigma_i(t)\Theta\left(\frac{q(t)}{N(t)} + \sqrt{\frac{q(t)}{N(t)}}\zeta_i(t) - \mu - \sigma\xi_i(t)\right) \\ &\approx N(t)\left\langle \Theta\left(\frac{q(t)}{N(t)} + \sqrt{\frac{q(t)}{N(t)}}\zeta(t) - \mu - \sigma\xi\right)\right\rangle_{(\zeta,\xi)} \\ &= N(t)\int dx\Theta\left(\frac{q(t)}{n(t)} - \mu + x\right)\rho(x|N(t)) , \end{aligned}$$

where we have defined

$$\rho(x|N(t)) \equiv \left\langle \delta\left(x - \sqrt{\frac{q(t)}{N(t)}}\zeta + \sigma\xi\right)\right\rangle_{(\zeta,\xi)} = \sqrt{\frac{1}{2\pi\left(\frac{q(t)}{n(t)} + \sigma^2\right)}} \exp\left[-\frac{x^2}{2\left(\frac{q(t)}{N(t)} + \sigma^2\right)}\right].$$

This, in turn, implies that:

$$N(t+1) \;\; = \frac{N(t)}{2}\left[\mathrm{erfc}\left(\frac{\mu - \frac{q(t)}{N(t)}}{\sqrt{2\left(\frac{q(t)}{N(t)} + \sigma^2\right)}}\right)\right].$$

Bifurcation analysis. A quick look at the naïve dynamical mean-field field equation suggests that we should expect two types of regimes in the parameter space: one in which the system is able to keep up with the external signal (the continuous $\&$ synchronous regime) and another one in which it does not (the continuous $\&$ asynchronous regime I) .

In order to identify this transition, we consider an exponentially decaying external signal $q(t) = q(0)e^{-t/\tau}$ henceforth. Let us first define $z(t) = \frac{\mu - T(t)}{\sqrt{2(\sigma^2 + T(t))}}$. If $z(t)$ were to be a constant, $z(t) = z_\star$, then, according to equation (4) we must have that $N(t) = N(0)\left(\frac{\mathrm{erfc}(z^\star)}{2}\right)^t = N(0)\left[\frac{\mathrm{erfc}(z_\star)}{2}\right]^t \equiv N(0)\exp\left[-t/\tau_n\right]$, where we have defined $\frac{1}{\tau_n} \equiv -\log\left[\frac{\mathrm{erfc}(z_\star)}{2}\right]$. On the other hand, since we are requiring $z_\star = z(t) = \frac{\mu - T(t)}{\sqrt{2(\sigma^2 + T(t))}}$ this automatically implies, as a matter of consistency, that $\tau = \tau_n$. Thus, for the region of the parameter space in which this identity holds, $N(t)$ and $q(t)$ do synchronize, and there are no avalanches. To identify in which part of the phase diagram a change of regime occur, we assume a continuous bifurcation to the region in which $\tau \neq \tau_n$. After some algebra we have the following set of coupled equations:

$$\begin{cases} e^{z_\star^2}\mathrm{erfc}\left(z_\star\right) = \frac{(\mu + 2(\kappa\mu)^2 + T_\star)T_\star}{2\sqrt{2\pi}((\kappa\mu)^2 + T_\star)^{3/2}} \\ z_\star(\tau) = \frac{\mu - T_\star}{\sqrt{2((\kappa\mu)^2 + T_\star)}} \end{cases}, \tag{4}$$

with $\frac{1}{\tau} = -\log\left[\frac{\mathrm{erfc}(z_\star)}{2}\right]$. Given μ, the solution of (4) results in a pair $(\kappa_c(\tau), T_\star(\tau))$. The first function $\kappa_c(\tau)$ corresponds to the bifurcation line separating both regimes. The line $T_\star(\tau)$ tells which value $T(t)$ takes precisely at the transition.

To check the validity of these results, we have simulated numerically eq. (4) and use the following parameter as a proxy for identifying the transition from the synchronous to the asynchronous regime:

$$\mathcal{O} = \lim_{T\to\infty} \frac{1}{T}\sum_{t=1}^{T} \left| \frac{n(t+1)}{n(t)} - e^{-\frac{1}{\tau}} \right| \quad (5)$$

This parameter has two drawbacks: First of all, it assumes a finite number of steps for the system to reach a quasi-stationary state, and secondly, it assumes that we can take a large enough time window. Clearly, these two assumptions will not be met when doing simulations. However, we can numerically compensate this when trying to identify the transition line by taking $\mathcal{O} < \epsilon$, for moderate values of ϵ. The corresponding line should be a lower bound of the analytical solution. A comparison between theory (dashed lines) and simulations is summarised in Figure 1 for three values of $\mu = 1$, $\mu = 100$ and $\mu = 1000$. Some comments are in order. First of all, we see that the comparison between the theory (4) and simulations is fairly good. Second of all, we note that, given a value of μ, there exists a value of τ, denoted τ_{gap}, below which there only exists the continuous regime. To find τ_{gap} as a function of μ, we simply put $\kappa = 0$ and solve the corresponding equations (4), obtaining:

$$\mu = -\frac{z_\star^2(\tau_{\mathrm{gap}})}{2} + 2\pi e^{2z_\star^2(\tau_{\mathrm{gap}})} \mathrm{erfc}^2[z_\star(\tau_{\mathrm{gap}})]\,. \quad (6)$$

This also implies that as $\mu \geq 0$, the gap has a maximum given by the solution:

$$z_\star(\tau_{\mathrm{gap}}^{(\mathrm{max})}) = 2\sqrt{\pi} e^{z_\star^2(\tau_{\mathrm{gap}}^{(\mathrm{max})})} \mathrm{erfc}[z_\star(\tau_{\mathrm{gap}}^{(\mathrm{max})})]\,, \quad (7)$$

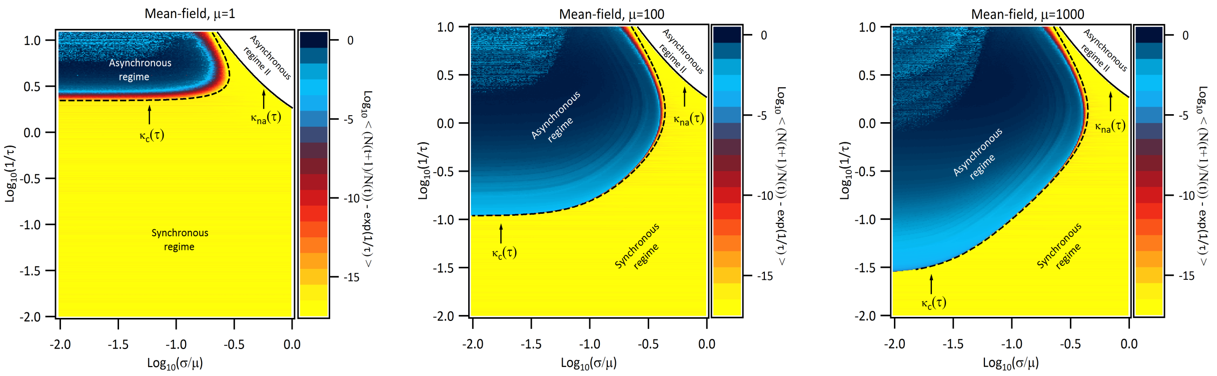

Figure 1: Phase diagram for the mean-field equation (4) for $\mu = 1$ (left), $\mu = 100$ (center) and $\mu = 1000$ (right).

which yields $1/\tau_{\text{gap}}^{(\text{max})} = 3.35229\cdots$. A plot of the gap as a function of μ can be found in Figure 2. Actually in the limit $\mu \to \infty$ we bifurcation line $\kappa_c(\tau)$ takes the following simpler form:

$$\kappa(\tau) = \frac{1}{\sqrt{2}} \frac{1}{\sqrt{\pi} e^{z_\star^2} \text{erfc}\,(z_\star) + z_\star(\tau)}\,. \tag{8}$$

As it turns out, there exists another region in the parameter space in which the system is not able

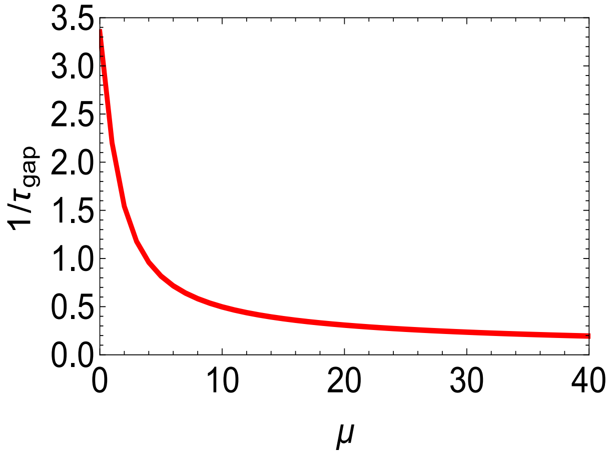

Figure 2: Left: Plot of $1/\tau_{\text{gap}}$ as function of μ.

to synchronize with the external signal, but this is due solely to the impossibility of the system to satisfy the conditions of the model. Indeed, notice that τ is an external parameter, while τ_n is given in terms of $z_\star$, that, in turn, depends on the value $T_\star$. This means that there exists a region in the parameter space in which $\tau \neq \tau_n$ as there is no initial conditions that will allow the system to realise $\tau = \tau_n$ after some transient. More precisely, we notice that:

$$\frac{1}{\tau} = -\log\left[\frac{1}{2}\mathrm{erfc}\left(\frac{1 - T_\star/\mu}{\sqrt{2(\kappa^2 + T_\star/\mu^2)}}\right)\right] \leq -\log\left[\frac{1}{2}\mathrm{erfc}\left(\frac{1}{\sqrt{2}\kappa}\right)\right] \equiv \frac{1}{\tau_{\mathrm{na}}}\,, \tag{9}$$

where we have used the fact that $T_\star$ and μ are both non-negative (at least in the context in which the model has been introduced). This naturally implies that above the bound $1/\tau_{\mathrm{na}}$ the corresponding region in the parameter space cannot realize the condition $\tau = \tau_n$ and $N(t)$ must decay more slowly than the external signal. This is what we call the continuous $\&$ asynchronous regime II. In the $(\kappa, 1/\tau)$-plane, the line $\kappa_{\mathrm{na}}(\tau)$ separating this region is thus given by:

$$-\log\left[\frac{1}{2}\mathrm{erfc}\left(\frac{1}{\sqrt{2}\kappa_{\mathrm{na}}}\right)\right] = \frac{1}{\tau}\,, \tag{10}$$

which is shown in Figure 1, as well as in Figure 3a on the main text.

Phase diagram of microscopic model As explained in the main text, to obtain the correct phase diagram we used Monte Carlo simulations of the microscopic dynamical equation (1) to identify the various phases in the parameter space $(\kappa, 1/\tau)$. As a proxy of inactivity over time, we have used the normalized cumulative time of inactivity. This is calculated by adding up the intervals Δt during which the activity remained unchanged, and subsequently dividing by the total duration of the activity, t_{TOTAL}. We consider the activity to last for a number t_{TOTAL}=$t_f - t_i$ of iterations,

where t_i is the first iteration for which $N(t_i + 1) \neq N(t_i)$, and t_f is the first iteration for which either $N(t_f) < 10$ or $T(t_f) < 10$. Figure 3 shows an example of the use of this metric for a particular time series.

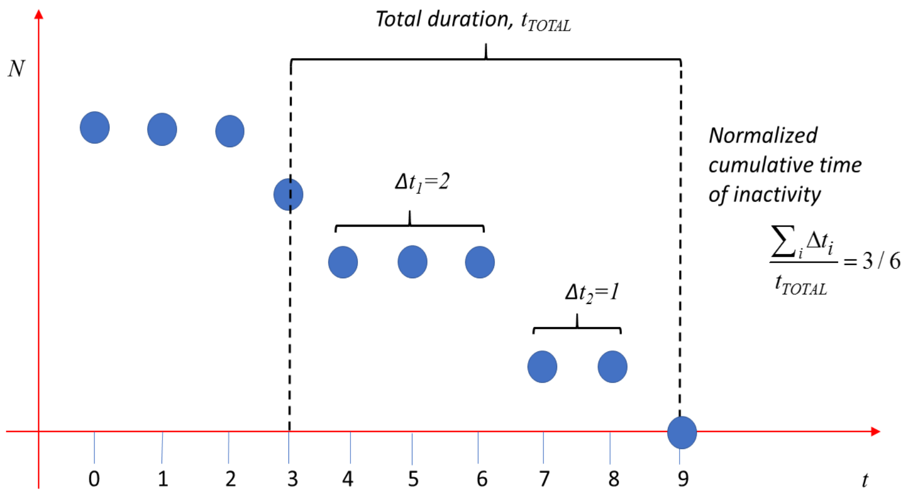

Figure 3: Pictorical representation to estimate the normalized cumulative time of inactivity.

2 Data analysis: comparison with real data

A data set consisting of both the time series for the weekly revenue and number of theaters of over 10,000 different movies was obtained from the website `www.boxofficemojo.com`. We kept only those 3469 that played in the U.S.A. for at least four consecutive weeks in no less than 50 theaters. The fit of the gross income to an exponential was in general excellent, with 90% of the correlation coefficients being higher than 0.98. An algorithm designed to fit noisy data was implemented that is able to signal when a change of dynamics has taken place so that only the portion of a given time series corresponding to the exponential decay was taken into account for the analysis. To obtain the number of people $q(t)$ that attended on a given week, the gross income per week for that movie was divided by the average ticket price for that year. The microscopic simulations were implementing using as initial conditions those found on the critical week t_c, defined as the latest week on which the movie was played in the maximum number of theaters. The reasoning behind this is the realization that new theaters will act as sources of exogenous shocks, which act as sources of new audience and may hinder the exponential relaxation of $q(t)$. Therefore, we expect that the system will relax without any further input once the number of theaters showing the movies does not increase anymore. Simulations of the microscopic model were performed for each movie using $q(0) = q(t_c)$ and $N(0) = N(t_c)$. Avalanche magnitudes, $N(t-1) - N(t)$ at every iteration t were normalized by the corresponding $N(0)$. The distribution obtained for the microscopic simulations were averaged from 100 different initial thresholds distributions for each time series. Further details about the algorithm mentioned above as well as about the fitting procedure and results can be found in reference[1].

1. Escobar, J. & Sornette, D. Dynamical signatures of collective quality grading in a social activity: attendance to motion pictures. *Plos One* **10**, 1 (2015).
